# Efficacy of EZH2 inhibitory drugs in human papillomavirus-positive and human papillomavirus-negative oropharyngeal squamous cell carcinomas

**DOI:** 10.1186/s13148-017-0390-y

**Published:** 2017-09-06

**Authors:** Cameron D. Lindsay, Morris A. Kostiuk, Jeff Harris, Daniel A. O’Connell, Hadi Seikaly, Vincent L. Biron

**Affiliations:** 1 Department of Surgery, University of Alberta, Edmonton, AB Canada; 2 Alberta Head and Neck Centre for Oncology and Reconstruction, Edmonton, AB Canada; 3 Department of Surgery, Division of Otolaryngology-Head and Neck Surgery, University of Alberta, Edmonton, AB Canada

**Keywords:** Histone methyltransferase, EZH2, Human papillomavirus, Oropharyngeal squamous cell carcinoma, Epigenetic

## Abstract

**Background:**

Head and neck squamous cell carcinoma (HNSCC) is the sixth most prevalent cancer worldwide with rates of HPV-positive oropharyngeal squamous cell carcinoma (OPSCC) dramatically increasing. The overexpression of enhancer of zeste homolog 2 (EZH2), a histone methyltransferase responsible for the trimethylation at lysine 27 of histone 3 (H3K27me3), is associated with a poor clinical prognosis and aggressive HPV-positive phenotypes.

**Methods:**

We utilized three EZH2 pathway inhibitors, GSK-343, DZNeP, and EPZ-5687, and tested their efficacy in two HPV-positive and two HPV-negative OPSCC cell lines.

**Results:**

Treatment with GSK-343 decreased H3K27me3 in all cell lines and treatment with DZNeP decreased H3K27me3 in only HPV-negative cell lines as determined by Western blot. Cells treated with EPZ-5687 displayed no appreciable change in H3K27me3. Epigenetic effect on gene expression was measured via ddPCR utilizing 11 target probes. Cells treated with DZNeP showed the most dramatic expressional changes, with decreased EGFR in HPV-positive cell lines and an overall increase in proliferation markers in HPV-negative cell lines. GSK-343-treated cells displayed moderate expressional changes, with CCND1 increased in HPV-positive cell lines and decreased TP53 in HPV-negative SCC-1. EPZ-5687-treated cell lines displayed few expressional changes overall. Only DZNeP-treated cells displayed anti-proliferative characteristics shown in wound-healing assays.

**Conclusions:**

Our findings suggest that EZH2 inhibitors are a viable therapeutic option for the role of epigenetic effect, potentially sensitizing tumors to current chemotherapies or limiting cell differentiation.

**Electronic supplementary material:**

The online version of this article (doi:10.1186/s13148-017-0390-y) contains supplementary material, which is available to authorized users.

## Background

Head and neck squamous cell carcinoma (HNSCC) is the sixth most prevalent cancer worldwide with over 600,000 new cases annually and 45,000 of those cases within North America alone [1]. Head and neck cancers are classified according to anatomic subsite, which include the oral cavity, oropharynx, hypopharynx, larynx, nasopharynx, and paranasal sinuses, with over 75% of HNSCCs attributed to the consumption of alcohol and tobacco products [2]. The incidence of HNSCCs overall has declined in many developed countries over the past 30 years; a trend attributed to public health campaigns. However, the incidence of oropharyngeal squamous cell carcinoma (OPSCC) has significantly increased over the past decade [3–5]. This increase in OPSCC is due to the exposure to high-risk serotypes of the human papillomavirus (HPV), with anywhere from 40 to 90% of OPSCC classified as HPV positive [4–9]. Clinical prognoses for HPV-positive OPSCCs are significantly better than HPV-negative cancers, as tumors display increased susceptibility to surgical intervention, chemotherapeutics, and radiotherapies [10–13]. These clinical differences are associated with distinct differences in HPV-positive OPSCC host gene expression and epigenetic profiles relative to HPV-negative cancers [14–20].

The field of epigenetics is defined as “the study of changes in organisms caused by modification of gene expression rather than alteration of the genetic code itself” [21]. Histone methylation, an epigenetic modification, has come increasingly to the forefront of cancer research, with evidence of dysregulation of various histone modifications such as trimethylation at lysine 20 of histone H4 and trimethylation at lysine 27 of histone H3 (H3K27me3) to be integral in the development and maintenance, metastasis, and resistance to chemo- and radiotherapies of various cancers [22–24]. EZH2, the catalytic component of the polycomb repressive complex 2 (PRC2), is responsible for H3K27me3 and has been shown to play a role in the development of prostate, breast, and head and neck squamous cell carcinomas. Overexpression of EZH2 has frequently been associated with increased tumor aggression and poor clinical outcomes, as well as a potential role in the formation of cancer stem cells [23–26]. This is believed to be due to the silencing effects of EZH2 on the expression of tumor suppressor genes [27].

HPV has shown to play a role in the aberrant expression of EZH2, as HPV viral oncoprotein E7 directly interacts with pathways responsible for EZH2 transcription, resulting in EZH2’s upregulation [17, 28]. p16(INK4A), a clinical surrogate marker for HPV positivity, further supported EZH2’s role in HPV-mediated tumorigenesis as a global trend of H3K27me3 elevation is present within the HPV-positive patient epigenome [18, 28]. Sartor and colleagues study [15] had also reported highly methylated promoter regions of PRC2 targets in HPV-positive cell lines relative to the HPV-negative variants. Given the evidence of EZH2’s potential for control of CpG methylation through direct physical contact with DNA methyltransferases (Dnmt) [29], EZH2 is an attractive chemotherapeutic target for both HPV-positive and HPV-negative OPSCC. We investigated the effects of three EZH2 pathway inhibitors: two S-adenosylmethionine (SAM)-competitive inhibitors (GSK-343 and EPZ-5687) and an S-adenosylhomocysteine (SAH)-hydrolase inhibitor (DZNeP) in HPV-positive (SCC-47 and SCC-104) and HPV-negative (SCC-1 and SCC-9) HNSCC cell lines. In this study, we demonstrated the inhibitors’ variable efficacies in reducing H3K27me3 as well as measured the epigenetic effect following treatment through gene expression changes.

## Methods

### Cell culture and drug treatment protocol

**Fig. 1:**
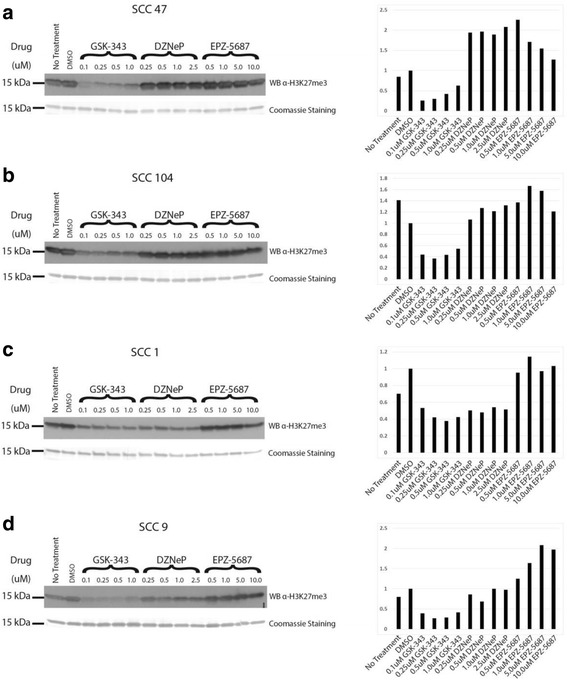
Drug effects on H3K27me3 levels vary between cell line’s HPV status. Western Blot analysis of H3K27me3 following 7-day treatments with GSK-343, DZNeP, or EPZ-5687 in **a** SCC47, **b** SCC104, **c** SCC1, **d** SCC9. *Left:* Western Blots with Coomassie *blue* staining (shown *below Western blot*) utilized as loading control. *Right:* Quantification of Western blot to *left* of graph based on fold differences to DMSO-treated cells

**Fig. 2:**
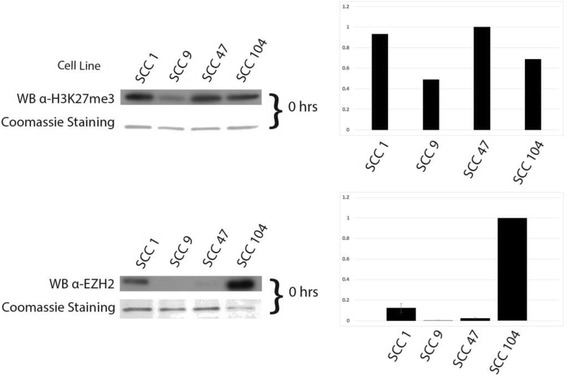
EZH2 and H3K27me3 baseline varies between cell lines. *Left*: Western Blot analysis of baseline EZH2 and H3K27me3 within individual cell lines. Coomassie blue staining (shown *below Western blot*) utilized as loading control. Right: Quantification of Western blot to *left* of graph based on fold differences to highest expressing cell line (EZH2 values versus SCC-104 expression, H3K27me3 values versus SCC-104 expression

**Fig. 3:**
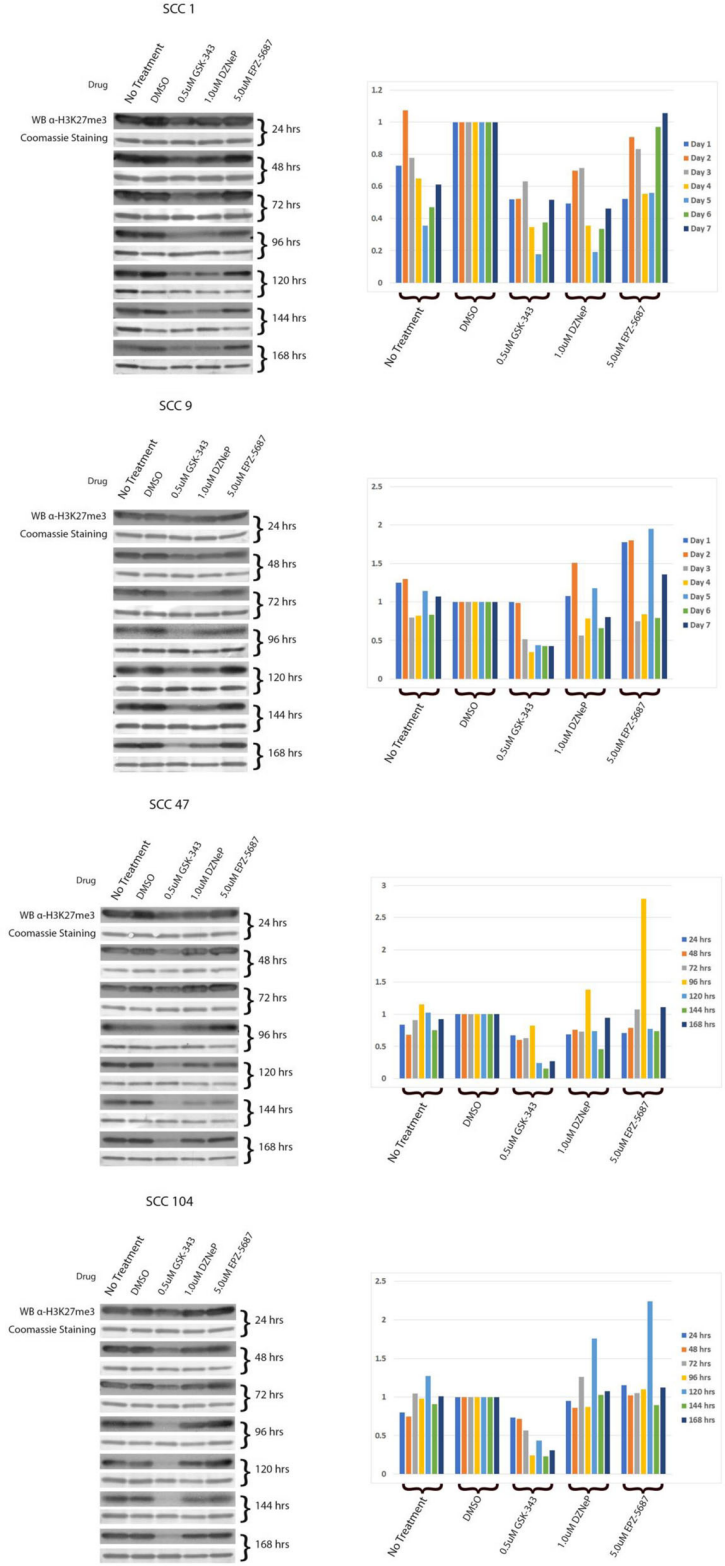
Inhibitor effects on methylation status occur as early as 48 h post treatment. *Left*: Western Blot analysis of H3K27me3. Coomassie *blue* staining (shown *below Western blot*) utilized as loading control. Cells were harvested and subjected to Western Blot analysis of H3K27me3 in 24 h intervals from 24 to 168 h (1–7 days) following the addition of an inhibitor (0.5 μM GSK-343, 1.0 μM DZNeP, or 5.0 μM EPZ-5687) at hour 0. *Right*: Quantification of Western blot to *left* of graph based on fold differences to DMSO-treated cells

**Fig. 4:**
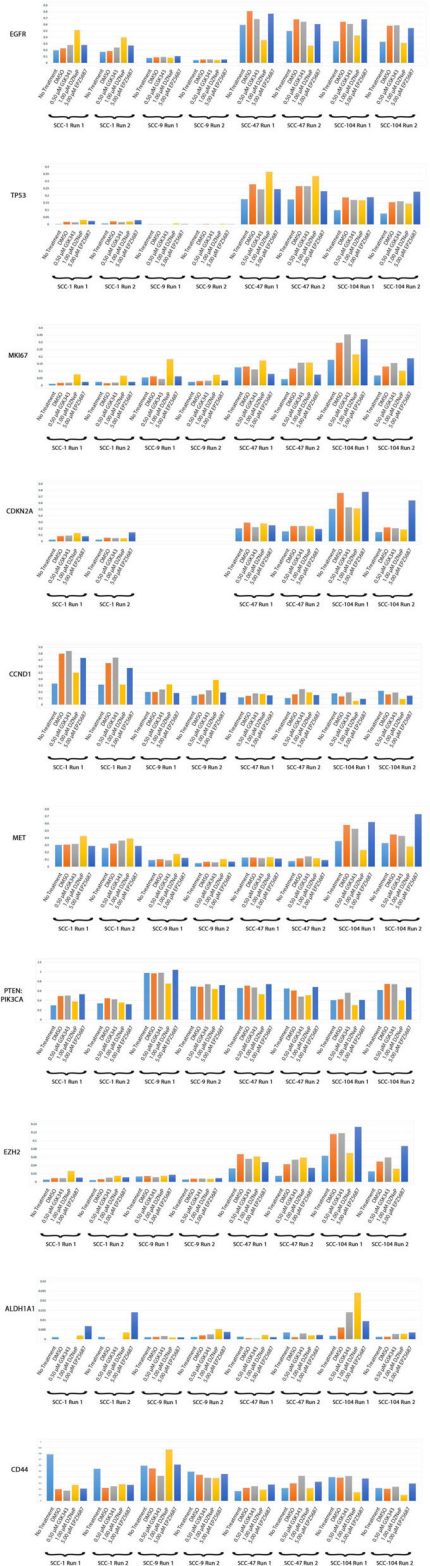
Treatment with inhibitors variably alters gene expression in all cell lines. Droplet digital PCR analysis of expressional ratios of nine target genes (EGFR, TP53, MKI67, CDKN2A, CCND1, MET, PTEN, PIK3CA, EZH2, ALDH1A1, and CD44) following 7-day treatment with GSK-343, DZNeP, or EPZ-5687. EEF2 was utilized as an internal reference, with exception to PTEN:PIK3CA as their gene products are directly antagonistic to one another. Scales vary according to individual expression results. SCC-9 cell line does not express CDKN2A and is therefore not pictured

**Fig. 5:**
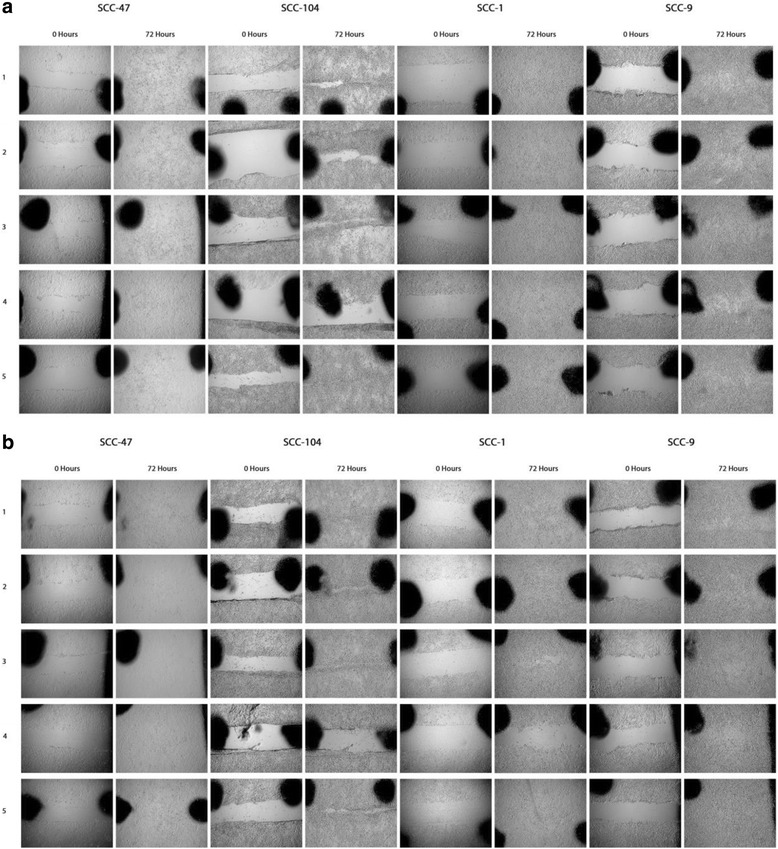
DZNeP displays anti-proliferative characteristics. Wound healing assay 0 to 72 h timeline with Row 1: No treatment, Row 2: 5.00 μM DMSO, Row 3: 0.50 μM GSK-343, Row 4: 1.00 μM DZNeP, or Row 5: 5.00 μM EPZ-5687. Images taken at 0 and 72 h. **a** Wound made same time as treatment with inhibitor. **b** Wound made 5 days following treatment with inhibitor

SCC-9 SCC-1, SCC-47, and SCC-104 cell lines were cultured using appropriate conditions [28, 30]. Cells were sub-cultured using common techniques with 0.25% Trypsin/EDTA and seeded at ~ 25% confluency with a 3-day settling and recovery period. On the third day, the cells were treated with fresh media containing the various inhibitors, DMSO alone, or media with no additives (no treatment). Cells were treated from 1 to 7 days depending on protocol, with fresh media and inhibitors added on day 4 when required. Day 7 Western blots utilizing inhibitor gradient (Fig. 1), Western blots of baseline EZH2 and H3K27me3 (Fig. 2), wound healing assays (Fig. 5), and Western blots of 7-day-treated H3K27me1 and H3K27me2 (Additional file 1: Figure S11 and Additional file 2: Figure S12, respectively) were performed three times (*n* = 3). Western blots measuring time of inhibition (Fig. 3) were performed once (*n* = 1) and ddPCR gene expression analysis (Fig. 4) was performed twice (*n* = 2).

### Wound-healing assay

Reference points 2 mm apart were made using an ultra-fine-tip sharpie on the underside of the culture plate prior to addition of cells. Following the recovery period, cells were either treated with various inhibitor concentrations or left untreated, depending on treatment group. Wound was made 5 days following recovery period, whereby cells were washed once with PBS and a 2 cm “wound” was made using a 1-mm-diameter ART10 pipette tip (cat:2139, ThermoScientific). Cells were then washed an additional two times with PBS to remove excess cellular debris, followed by the addition of appropriate media and treatment. Digital photographs of the wound were taken at 50× magnification using the ZEISS Stemi 2000-C Stereo Microscope mounted with AxioCam ERc5s (ZEISS) and developed using ZEN 2011 Imaging software (ZEISS). Photos were taken at 12-h intervals for 72 h. Media and inhibitor treatment was changed every 24 h prior to photographs to ensure wound clarity. Contrast and sharpness of images were corrected manually for purposes of clarity utilizing Photoshop CC (Adobe Systems).

### Whole cell lysate and Western blot

Whole cell lysates were utilized in the analysis of EZH2 protein content and were obtained from harvesting cells in 200 μL 50 mM TRIS +1% SDS pH 7.6, supplemented with 1 mM phenylmethylsulfonyl fluoride (PMSF) and 1 × complete protease inhibitor (CPI) (Cat#: 11697498001 Roche). Cells were incubated on ice for 20 min, sheared through a 25-gauge syringe, and centrifuged for 15 min at 16,000 ×*g* at 4 °C. One hundred milliliters of supernatant was transferred to new centrifuge tubes and was either stored at − 20 °C or subjected to SDS-PAGE and Western blot. A target of 15 μg of protein per well was used for SDS-PAGE. Following transfer, polyvinylidene fluoride (PVDF) membranes were blocked overnight at 4 °C in phosphate buffered solution in a 0.1% Tween20 in PBS (PBST) + 5% milk solution. Membranes were incubated for 1 h at ambient temperature with a 1:1000 dilution of both anti-EZH2 primary antibody (#5246 Cell Signalling Technology) in PBST + 5% milk solution. Membranes were washed extensively in PBST and incubated separately in 1:5000 HRP-conjugated anti-rabbit secondary antibody (Cat#: 170-5046 BIO-RAD) and 1:5000 HRP-conjugated anti-mouse secondary antibody (Cat#: 170-5047 BIO-RAD) for 1 h at room temperature in PBST + 5% milk. Membranes were washed extensively with PBST and treated with Amersham ECL Prime Western Blotting Detection Reagent kit (Cat#: RPN2236, GE Healthcare) to reveal chemiluminescence prior to exposure to film (Fugifilm Medical XRAY Film Super RX). PVDF membranes were then washed extensively in hot water and subjected to a 20-s incubation in a 45% methanol and 10% acetic acid Coomassie Blue R-250 solution (Cat#: 161-0400 BIO-RAD). Membranes were washed again with hot water, patted dry, and allowed to dry further overnight.

### Histone enrichment and Western blot

Histones were enriched from cell lysates following the Abcam protocol “Histone extraction protocol for western blot” (reference http://www.abcam.com/protocols/histone-extraction-protocol-for-western-blot) with minor changes. Following incubation in 0.2 N HCL, tubes were briefly vortexed, then centrifuged for 10 min at 6500 ×*g* at 4 °C. The histone-containing supernatant (~ 400 μl) was transferred to new tubes and 100 μl of Trichloroacetic acid was added. The tubes were vortexed briefly and incubated on ice for 1.5 h followed by centrifugation for 15 min at 16,000 ×*g* and 4 °C. The supernatant was aspirated and the pelleted protein was washed twice with − 20 °C acetone and allowed to dry with the lid open at ambient room temperature for 1.5 h. Pelleted histones were either stored at − 20 °C or immediately resuspended in 0.1 M NaOH and subject to SDS-PAGE and Western blot. A target of 5 μg of protein per well was used for SDS-PAGE. Following transfer, polyvinylidene fluoride (PVDF) membranes were blocked overnight at 4 °C in phosphate buffered solution in a 0.1% Tween20 in PBS (PBST) + 5% milk solution. Membranes were incubated for 1 h at ambient temperature with a 1:1000 dilution of anti-H3K27me3 primary antibody (ab6002 Abcam) in PBST + 3% milk solution. Membranes were washed extensively in PBST and incubated in 1:5000 HRP-conjugated anti-mouse secondary antibody (Cat#: 170-5047 BIO-RAD) for 1 h at room temperature in PBST + 5% milk. Membranes were washed extensively with PBST and treated with Amersham ECL Western Blotting Detection Reagent kit (Cat#: RPN3243, GE Healthcare) to reveal chemiluminescence prior to exposure to film (Fugifilm Medical XRAY Film Super RX). Other target proteins of interest utilized an identical protocol as above. A 1:100 dilution of anti-H3K27me2 primary antibody (ab194690 Abcam) in PBST + 5% milk solution was used to identify H3K27me2 expression following treatment (Additional file 2: Figure S12), followed by an incubation in 1:2000 HRP-conjugated anti-rabbit secondary antibody (Cat#: 170-6515 BIO-RAD) in PBST + 5% milk. A 1:4000 dilution of anti-H3K27me1 primary antibody (ab194688 Abcam) in PBST + 5% milk solution was also used to identify H3k27me1 expression levels following treatment (Additional file 1: Figure S11), this was followed by an incubation in 1:20,000 HRP-conjugated anti-rabbit secondary antibody (Cat#: 170-6515 BIO-RAD) in PBST + 5% milk. PVDF membranes were washed extensively in hot water and subjected to a 20-s incubation in a 45% methanol and 10% acetic acid Coomassie Blue R-250 solution (Cat#: 161-0400 BIO-RAD). Membranes were washed again with hot water, patted dry, and allowed to dry further overnight.

### Western blot quantification and analysis

Quantification of Western blots was performed using ImageJ (v. 1.51j8 bundled with Java v. 1.8.0_112 National Institutes of Health) on scans of exposed films and Coomassie-stained PVDF membranes. Scans were performed at 600DPI using an EPSON XP-610 All-In-One Printer (C11CD31201 Epson). Scans with lowest exposure while maintaining visible band differentiation were chosen for quantification. Values were calculated according to mean gray value of band and standardized via subtraction of nearby background. Gray value of standardized blots were normalized through division by a common, standardized Coomassie-stained band. Quantification area was maintained between band and subtracted background. Ratios were obtained by dividing treatment group values by either the highest expression values or DMSO-only values.

### RNA extraction, purification and ddPCR

Cells were washed with 3 × 2 ml PBS, scraped, and transferred in 300 μL RNA Later (AM7021 ThermoScientific) to 2-ml centrifuge tubes. The tubes were vortexed, and 20 μL of sample was transferred to a new centrifuge tube. RNA purification was performed using the RNeasy Plus Mini Kit supplemented with gDNA Eliminator mini Spin Columns (Cat#: 74,134 Qiagen) and QIAshredder (Cat#: 79,656 Qiagen). Forty nanograms of RNA was used to synthesize complementary DNA (cDNA) using the iScriptTM Reverse Transcription Supermix for RT-qPCR (Cat#: 1708841 BIO-RAD) as per the manufacturer’s protocol. Following the reaction, the cDNA was diluted with Nuclease-free H2O to 1 ng/μl and either stored at − 20 °C or used directly for ddPCR as described previously [31]. Primers/probes utilized are as follows: EGFR (unique assay ID: dHsaCPE5038080/dHsaCPE5038081 BIO-RAD), TP53 (unique assay ID: dHsaCPE5037520/dHsaCPE5037521 BIO-RAD), MKI67 (unique assay ID: dHsaCPE5050322/dHsaCPE5050323 BIO-RAD), CDKN2A (unique assay ID: dHsaCPE5045104/dHsaCPE5045105 BIO-RAD), CCND1 (unique assay ID: dHsaCPE5051730/dHsaCPE5051731 BIO-RAD), MET (unique assay ID: dHsaCPE5034172/dHsaCPE5034173 BIO-RAD), PTEN (unique assay ID: dHsaCPE5030136/dHsaCPE5030137 BIO-RAD), PIK3CA (unique assay ID: dHsaCPE5058352/dHsaCPE5058353 BIO-RAD), EZH2 (unique assay ID: dHsaCPE5034224/dHsaCPE5034225 BIO-RAD), EEF2 (unique assay ID: dHsaCPE5050048/dHsaCPE5050049 BIO-RAD), ALDH1A1 (unique assay ID: dHsaCPE5056918/dHsaCPE5056919 BIO-RAD), and CD44 (unique assay ID: dHsaCPE5051600/dHsaCPE5051601 BIO-RAD). Where an internal reference for gene expression was required, human EEF2 primers/probe were used. GAPDH primers/probe (unique assay ID: dHsaCPE5031596/dHsaCPE5031597 BIO-RAD) were utilized as a secondary internal reference and compared to EEF2 values against a probe with known trends, EGFR (See Additional file 3: Figure S10). Reactions were set up in a 96-well plate according to the QX200 Droplet Generator Instruction Manual (Cat#: 10031907 BIO-RAD).

## Results

### Drug effects on H3K27me3 levels vary between cell line’s HPV status

To determine the individual drug efficacy of EZH2 inhibition, Western blot analysis was utilized to detect changes in the levels of the EZH2 catalysis product, H3K27me3. This was performed on individual cell lines with an anti-H3K27me3 monoclonal antibody (Fig. 1).

Treatment with GSK-343 displayed a clear reduction of H3K27me3 that was consistent in all cell lines (Fig. 1). In contrast, inspection of Western blots shows clear differences in H3K27me3 reduction based on HPV status in the SAH-hydrolase inhibitor DZNeP-treated cells. Analysis of the Western blots show DZNeP-treated cell lines have a reduction of H3K27me3 only present in HPV-negative cell line SCC-1, while HPV-negative SCC-9 and HPV-positive cell lines (SCC-47 and SCC-104) appear to have H3K27me3 levels comparable to untreated or DMSO only-treated cells. This reduction in H3K27me3 is also not as dramatic when compared to GSK-343-treated cell lines (Fig. 1c, d). Treatment with the other SAM-competitive inhibitor, EPZ-5687, resulted in no apparent demethylation in any cell lines, with H3K27me3 levels comparable to DMSO or untreated cells.

### EZH2 and H3K27me3 baseline varies between cell lines, with inhibitor effects on methylation status occurring as early as 48 h post treatment

To compare the baseline protein levels of EZH2 and H3K27me3 in individual cell lines, Western blot analysis was performed utilizing either an anti-EZH2 or anti-H3K27me3 antibody (Fig. 2). Both baseline EZH2 and H3K27me3 status showed clear variability between individual cell lines. However, this variability appears to contradict what one would expect, that being higher EZH2 levels would result in higher levels of H3K27me3 [23]. HPV-negative SCC-9 is the caveat to this, as it displays the lowest amount of EZH2 and H3K27me3 expression relative to other cell lines. HPV-positive SCC-104 displayed moderately elevated levels of H3K27me3, while its EZH2 expression displays the highest amount of EZH2 relative to the other cell lines utilized. SCC-1 and SCC-47 show the highest levels of H3K27me3 relative to the other cell lines; however, HPV-positive SCC-47 displays low EZH2 expression and HPV-negative SCC-1 displays high EZH2 expression.

The timeline of drug effect was determined via Western blotting analysis utilizing an anti-H3K27me3 monoclonal antibody as above. Cell lines were treated with midline concentrations of GSK-343, DZNeP, or EPZ-5687 (Fig. 3). Endpoint (168 h) Western blotting results remained consistent with the results presented in Fig. 1. All GSK-343-treated cell lines displayed decreased H3K27me3. DZNeP-treated cell lines displayed decreased H3K27me3 in HPV-negative SCC-1 cell line. EPZ-5687-treated cell lines displayed no change in H3K27me3. GSK-343-treated cell lines displayed the most immediate drug effects in all cell lines, with demethylation occurring between 24 and 48 h post treatment and maximum H3K27me3 reduction occurring at 96 h post treatment. DZNeP-treated SCC-1 displayed demethylating effects later than GSK-343-treated cell lines, with demethylation first appearing between 48 and 72 h post treatment and maximum H3K27me3 reduction at 144 h post treatment. Again, EPZ-5687 had no appreciable reduction in H3K27me3, with levels remaining comparable to DMSO or untreated cells. There are apparent reductions in H3K27me3 levels at 120 and 144 h in DZNeP- and EPZ-5687-treated SCC47 cells. However, when observing drug effect timeline patterns observed in other cell lines, combined with 7-day post-treatment H3K27me3 levels returning to repeatable levels, these reductions are most likely the result of random artifact and not drug effect.

### Treatment with inhibitors variably alters gene expression in all cell lines

Numerous gene products have been shown to have direct oncogenic properties, or have shown a high degree of correlation in OPSCC. Using droplet digital polymerase chain reaction (ddPCR) in conjunction with reverse transcriptase PCR (RT PCR), a selection of 11 different genes (EGFR, TP53, MKI67, CDKN2A, CCND1, MET, PIK3CA, PTEN, EZH2, ALDH1A1, and CD44) frequently associated with HPV-positive and HPV-negative OPSCC were quantified for expressional changes against an internal normalization control EEF2 (Fig. 4, Additional file 4: Figure S9). Reported results were noted according to differences and trends that deviated from DMSO-treated cells in both runs.

GSK-343-treated cell lines displayed only moderate changes in gene expression of targeted genes. A discriminatory trend based on HPV status was also identified. Of the observable changes, HPV-positive SCC-47s and SCC-104s showed an upward trend of CCND1 expression following treatment with increasing GSK-343 concentrations. Of the HPV-negative cell lines, only SCC-1 showed a slight decreasing trend in TP53 expression following treatment with increasing concentrations of GSK-343. SCC-9 showed no clear changes following treatment with GSK-343.

Treatment with DZNeP displayed the greatest variability and overall amount of expressional changes within the cell lines relative to GSK-343 and EPZ-5687. A slight discriminatory trend based on HPV status was also identified. HPV-positive SCC-47 and SCC-104 both displayed a downward trend in EGFR expression with increasing DZNeP concentrations. SCC-47 also showed slightly elevated TP53 that increased with increasing DZNeP concentrations and SCC-104 showed static decreases in CCND1, MET, CD44, EZH2, and PTEN:PIK3CA following treatment with DZNeP. The HPV-negative cell line SCC-1 displayed an upward trend of increasing EGFR and TP53, slight increases in MET and CDKN2A, and an increase in MKI67 that followed a downward trend with increasing DZNeP concentration. SCC-1 cells also saw a static decrease in CCND1 following treatment with DZNeP. HPV-negative SCC-9 cells displayed static increases in TP53, MKI67, MET, and CCND1, as well as a static decrease in PTEN:PIK3CA following treatment with DZNeP.

EPZ-5687-treated cell lines displayed few expressional changes in both HPV-positive and HPV-negative cell lines. SCC-47 cells showed downward trend of decreased MKI67 expression, and a slight downward trend of decreased CCND1 was seen in SCC-104 cells with increasing EPZ-5687 concentration. A static increase in CDKN2A was seen in HPV-negative SCC-1 cells following treatment with EPZ-5687.

Of note, treatment with DMSO alone altered gene expression of many target genes relative to cells that remained untreated. The most dramatic of these changes were seen in the HPV-positive cell lines (SCC-47 and SCC-104). DMSO-treated SCC-47 cells showed an increased expression in target gene markers EGFR, TP53, MKI67, CDKN2A, CCND1, MET, and EZH2 while PTEN:PIK3CA, CD44, and ALDH1A1 expression remained comparable to untreated cell lines. DMSO-treated SCC-104 cells displayed an increase in target genes EGFR, TP53, MKI67, CDKN2A, MET, PIK3CA, PTEN, EZH2, ALDH1A1, and CD44. CCND1 expression had decreased following treatment with DMSO. HPV-negative SCC-1 cells showed increased expression in TP53, CDKN2A, CCND1, and PTEN:PIK3CA and decreased levels of CD44 following treatment with DMSO alone. SCC-9 cells showed no appreciable changes following treatment with DMSO.

### DZNeP displays anti-proliferative characteristics

To determine anti-proliferative effects of the inhibitors, as well as establish an approximate timeline of said effects, wound-healing assays were performed with drug application made at two separate time points. The first time point included the addition of the inhibitors on the same day the wound was made (Fig. 5a). The second time point included the addition of inhibitors 5 days prior to the wound being made (Fig. 5b). Of note, SCC-9 cell lines include a transformed fibroblast feeder layer with variably dispersed tumor foci [29]. Due to the nature of the SCC-9 cell line, observed behavior is likely more representative of this fibroblast layer rather than cancerous cells.

Wounds made on the same day as treatment (Fig. 5a) had closed by 72 h with no evidence of drug effects on cell proliferation. DZNeP-treated SCC-104 cells initially appear to contradict this statement, as wounds remained open after the 72-h period. The self-adherent properties of the cell line can be attributed to this result, as the observed rate of cell proliferation in the same day-treated cells far exceeded cells pre-treated with DZNeP 5 days prior to wound being made (Fig. 4b). SCC-47 cells with treatment made on the same day as wound had closed by 24 h. DMSO- and EPZ-5687-treated cells wound remained open for a longer duration relative to other treatments (Additional file 5: Figure S1; untreated, GSK-343, and DZNeP-treated cell lines had all closed by 12 h). This difference can most likely be attributed to the slightly larger wound margin, as 5-day DMSO and EPZ-5687 pre-treated cells had closed by a comparable timeline to the untreated cells (Additional file 6: Figure S5). These results are comparable to treated SCC-1 cell lines (Additional file 5: Figure S3), with all but the untreated group closing by 36 h. The results of untreated SCC-1 cells can also be attributed to a wider wound margin, as their 5-day “pre-treated” counterparts (Additional file 6: Figure S7) close at an earlier time point of 48 h.

All cell lines treated with DZNeP 5 days prior to wound being made (Fig. 5b) displayed clear differences relative to those made on the same day (Fig. 4a), with wounds remaining open after 72 h in SCC-104, SCC-1, and SCC-9 cells. DZNeP-treated SCC-47 cells had also displayed decreased proliferation; however, wound closure occurred between 24 and 36 h (Additional file 6: Figure S5). GSK-343-treated SCC-1 cells had impartial wound closure after 72 h, suggesting slight anti-proliferative properties. All other cell lines had complete wound closure by 72 h. All EPZ-5687-treated cell lines wounds had complete wound closure by 72 h with timelines comparable to DMSO alone. DMSO appeared to display anti-proliferative properties in the SCC-1 cells, with wound closure occurring at a later time point (Additional file 6: Figure S7; between 48 and 60 h) than the untreated group (between 36 and 48 h).

## Discussion

This study is a continuation of our previous work in 2016 [19], with the aim of further expanding our investigation of EZH2 inhibition as a potential chemotherapeutic target for use in OPSCC. With the incidence of HPV-positive OPSCC rising [5, 6], the need for targeted therapies becomes increasingly urgent. Given the elevated expression of EZH2 in aggressive HPV-positive OPSCC phenotypes as well as other metastatic cancers, EZH2 and its related pathways remain an attractive target for chemotherapeutic agents. Our investigation of three epigenetic inhibitors, GSK-343, DZNeP, and EPZ-5687, suggests targeting of EZH2 pathways may be of therapeutic benefit in OPSCC. EPZ-5687, however, does not appear to be an effective inhibitor of EZH2 in OPSCC.

DZNeP has been shown by others to be effective as an anti-tumoral agent alone or when combined with other agents [32–34]. DZNeP is one of the first known inhibitors of EZH2 and has well documented epigenetic and anti-proliferative effects [33]. Our findings further support these properties. DZNeP is a small-molecule SAH-hydrolase inhibitor that has been shown to act as a global histone methyltransferase (HMT) inhibitor [35]. When used alone as treatment, DZNeP has shown anti-tumor activity in multiple cancer types including breast [36], brain [37], liver [38], lung [39], and prostate [40]. Leukemia and prostate models have demonstrated DZNeP’s anti-proliferative [41] and anti-metastatic [40] properties, respectively, and reduced tumor-mitigated angiogenesis in a glioblastoma xenograft model [42]. Our findings suggest its potential to decrease the expression of known HNSCC stem cell marker CD44 [43]. Unfortunately, in vivo pharmacological studies have demonstrated DZNeP to have a short half-life [44] and high toxicity in animal models at higher concentrations [33, 35]. However, gene expression results in our findings display a relatively static trend in all cell lines. This would suggest epigenetic effect to occur in concentrations lower than 0.25 μM and potentially lowering cytotoxic properties of the inhibitor. Another interesting result obtained following treatment with DZNeP is the variable H3K27me3 levels with variable efficacy based on HPV status. However, expressional data suggests epigenetic effect still remains present in all cell lines. This discrimination may be due to potential interactions of HPV viral proteins with DZNeP or its related pathways in combination with DZNePs effects on histone methylation to be global rather than EZH2 specific [35], leaving the desired epigenetic effect to carry out via an alternate pathway.

GSK-343 treated cells displayed H3K27me3 reduction in all cell lines, with no apparent resistance or sensitivity seen with application of DZNeP. The changes in gene expression as a result of GSK-343 were moderate relative to DZNeP, with evidence of dose-dependence and discrimination based on HPV status. These changes could be reflective of the mechanism of action of GSK-343 as an S-adenosyl-L-methionine (SAM)-competitive inhibitor of EZH2, and its increased specificity to EZH2 alone relative to DZNeP. The observed dose-dependent nature of GSK-343 may also play a role in the moderate expression results observed. Previous studies have shown GSK-343 treatments result in significant epigenetic effects in breast cancer, colon cancer, and leukemia cell lines [45], as well as inducing autophagy in hepatocellular carcinomas [46]. Treatment with GSK-343 has also been shown to induce phenotypic reprogramming of cervical cancer cell lines from mesenchymal to epithelial both in vitro and in vivo [47]*.* This reprogramming observed reduced cell proliferation and motility, thereby blocking tumor invasion to nearby tissue. Given the cellular commonalities shared between cervical and OPSCC [48] as well as evidence of epigenetic effect seen in our study, GSK-343 remains a promising agent for combined use with anti-proliferative therapies.

EPZ-5687 showed very little efficacy in both the ability to demethylate H3K27me3 or lead to desired expressional changes of observed genes within an OPSCC model. These results were unexpected given EPZ-5687 is also an S-adenosyl-L-methionine (SAM)-competitive inhibitor of EZH2 and has shown > 500-fold selectivity to EZH2 over other human protein methylases in lymphoma models [49]. The possibility of this discrimination could be due to EPZ-5687’s > 5-fold affinity to the A677G mutant over EZH2 wildtype, but H3K27me3 inhibition was still present in both variants. However, the Pfeiffer cell line utilized in Knutson and colleagues study showed a much greater sensitivity to EPZ-5687 relative to others they had utilized [49], suggesting the potential of secondary factors to contribute to EPZ-5687’s efficacy of inhibition. Unfortunately, very little published information is available on this inhibitor to identify any consistent trends.

At the transcriptional level, HPV status appeared to play a deterministic role in gene expressional changes as a result of treatment with the EZH2 inhibitors observed. This discrimination in expressed genes may be a result of the differential DNA methylation profiles displayed in HPV-positive carcinomas [15, 50, 51]. Theoretically, varying methylation at promotor regions may limit the epigenetic effects of EZH2 inhibitors, instead only reactivating genes not silenced by DNA methylation. A study performed by Bartke and colleagues provided evidence toward the limitations of histone methyltransferase and DNA methyltransferase (Dnmt) inhibitors alone by demonstrating reduced regulation of PRC2 following DNA methylation [52]. They observed a far more complex interaction between histone and DNA methylation statuses on transcriptional activity, involving deregulation of one enzyme based on methylation status of DNA or histone. Therefore, desired epigenetic effects of EZH2 inhibitors may be further enhanced by combination with Dnmt inhibitors such as 5-azacytidine. Proposed combinations such as this, of course, run the risk of increasing toxic effect. DNA demethylating agents have traditional anti-proliferative activity at high doses. 5-Azacytidine, for example, has been shown to cause neutropenia [53]. Fortunately, demethylating agents have been shown to be generally mild at lower doses [54] and for the proposed epigenetic action, the use of extremely low doses for both agents (EZH2 inhibitors and Dnmt inhibitors) could be utilized to avoid this anti-proliferative activity. Our study further supports this notion, as expressional changes occurred in both GSK-343 and DZNeP at low doses. ChIP-sequence analyses performed on colon, breast, and leukemia cancer cell lines by Sato and colleagues [45] has already shown evidence for the synergistic effects of Dnmt inhibitors with histone methyltransferase inhibitors while maintaining their selectivity toward various oncogenes.

The most prominent limitation of this study is seen in the expression results following treatment with DMSO. The majority of targeted genes in both HPV-negative SCC-1 and HPV-positive SCC-47 and SCC-104 have displayed clear alterations in gene expression following treatment with DMSO as compared to untreated cells. Historically, DMSO has been shown to have multiple effects on cellular functioning [55]. Its most noteworthy trait includes its ability to induce differentiation in malignant tumor cells at low doses, resulting in a loss of tumorigenicity and altered cell morphology into more mature, differentiated cells in leukemias, colon, prostate, lung, and breast carcinomas [56–58]. The exact mechanism of DMSO’s ability to induce differentiation remains relatively unknown; however, a study performed by Iwatani et al. suggests DMSO impacting a cell’s epigenetic profile. Their study had shown the upregulation of DNA methyltransferase Dnmt3a and alteration of genome-wide DNA methylation profiles following the application of DMSO [59]. Contrasting results were seen in a study performed by Kita et al., showing DMSO reducing stemness in association with decreased DNMT3A and DNMT3L expression [60]. Regardless of the contention, genome-wide methylation changes would theoretically be present and it is understandable why our gene target expression levels deviated from untreated baseline levels so dramatically. Given DMSOs’ already widespread clinical use as a drug vehicle and cryopreservant, as well as the limited amount of solvents available for water-insoluble drug agents, it is an issue that will continue to persist. Additionally, there are inherent limitations of quantifying values from film paper, as overexposure of film runs the potential of film “reverse banding” or “photobleaching”, potentially providing lower values in the quantification process than what is representative. The opposite is true as well, with saturated dark values being limited to the darkening capacity of the film following reaction from silver halide molecules [61, 62]. To minimize these issues, lower exposure times were utilized in the quantification process. Other limitations of this study include the use of in vitro models on only four cell lines.

Both DZNeP and GSK-343 display potential for clinical application as adjunctive therapies in OPSCC, potentially sensitizing tumors to current chemotherapies or limiting cell differentiation. Previous research suggests the use histone modification inhibitors in combination with Dnmt inhibitors could have synergistic effects on epigenetic changes within the cell [45, 63–66]. Future experimentation warrants the use of primary cell cultures as well as tumor xenografts models to further determine clinical efficacy.

## Conclusions

In HNSCC cell culture models, the targeting of EZH2 and its related pathways appears to have anti-tumorigenic effects which may be dependent on oncogenic HPV status. EPZ-5687 does not appear to be an effective inhibitor of EZH2 in both HPV-positive and HPV-negative HNSCC cell lines.

## Additional files

Additional file 1: **Supplemental Figure 11.** Monomethylated H3K27 levels do not appear to change following treatment with inhibitors. (PDF 17191 kb)

Additional file 2: **Supplemental Figure 12.** Dimethylated H3K27 levels do not appear to change following treatment with inhibitors. (PDF 17232 kb)

Additional file 3: **Supplemental Figure 10.** EGFR displayes comparable trends when both EEF2 and GAPDH are utilized as internal controls. (PDF 10631 kb)

Additional file 4: **Supplemental Figure 9.** Treatment with inhibitors variably alters gene expression in all cell lines. (ZIP 148366 kb)

Additional file 5: **Supplemental Figures 1-4.** Wound healing assay 0 hour to 72 hour timeline all time points. Wound made same day as treatment with inhibitor. (PDF 118729 kb)

Additional file 6: **Supplemental Figures 5-8.** Wound healing assay 0 hour to 72 hour timeline all time points. Wound made 5 days after treatment with inhibitor.(PDF 119715 kb)

## Abbreviations

Bp: Base pair
DMSO: Dimethylsulfoxide
DNA: Deoxyribonucleic acid
Dnmt: DNA methyltransferase
DZNep: 3-Deazanoplanocin
EZH2: Enhancer of zeste homolog 2
H3K27me3: Histone H3 trimethylation of lysine 27
HAT: Histone acetyltransferase
HDAC: Histone deacetylase
HDM: Histone demethylase
HMT: Histone methyltransferase
HNSCC: Head and neck squamous cell carcinoma
HPV: Human papillomavirus
OPSCC: Oropharyngeal squamous cell carcinoma
PBS: Phosphate buffered saline
PBS-T: Phosphate buffered saline + tween20
PRC2: Polycomb repressive complex 2
PVDF: Polyvinylidene fluoride
RNA: Ribonucleic acid
SAM: S-Adenosyl-L-methionine
