# Supplementary material for: Efficacy of EZH2 inhibitory drugs in human papillomavirus-positive and human papillomavirus-negative oropharyngeal squamous cell carcinomas
Source: Clin Epigenetics. 2017 Sep 6;9:95. doi: 10.1186/s13148-017-0390-y (PMC5586065; doi:10.1186/s13148-017-0390-y)
Supplement: Supplementary file 3 — Supplemental Figure 10. EGFR displayes comparable trends when both EEF2 and GAPDH are utilized as internal controls. (PDF 10631 kb) [file 13148_2017_390_MOESM3_ESM.pdf]

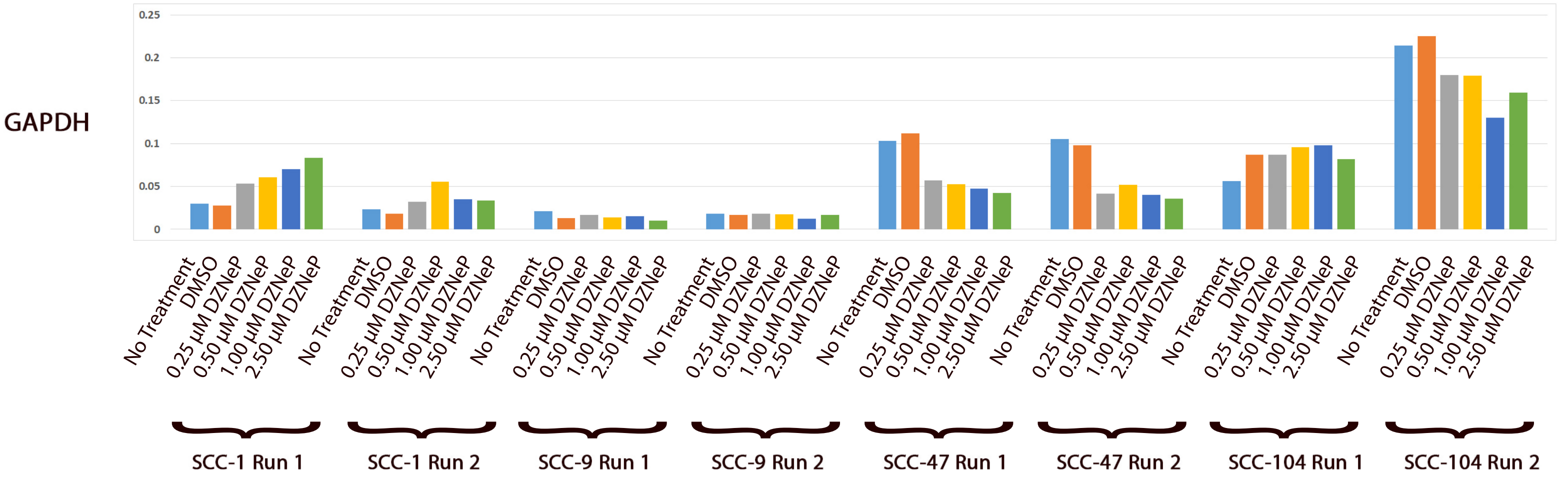

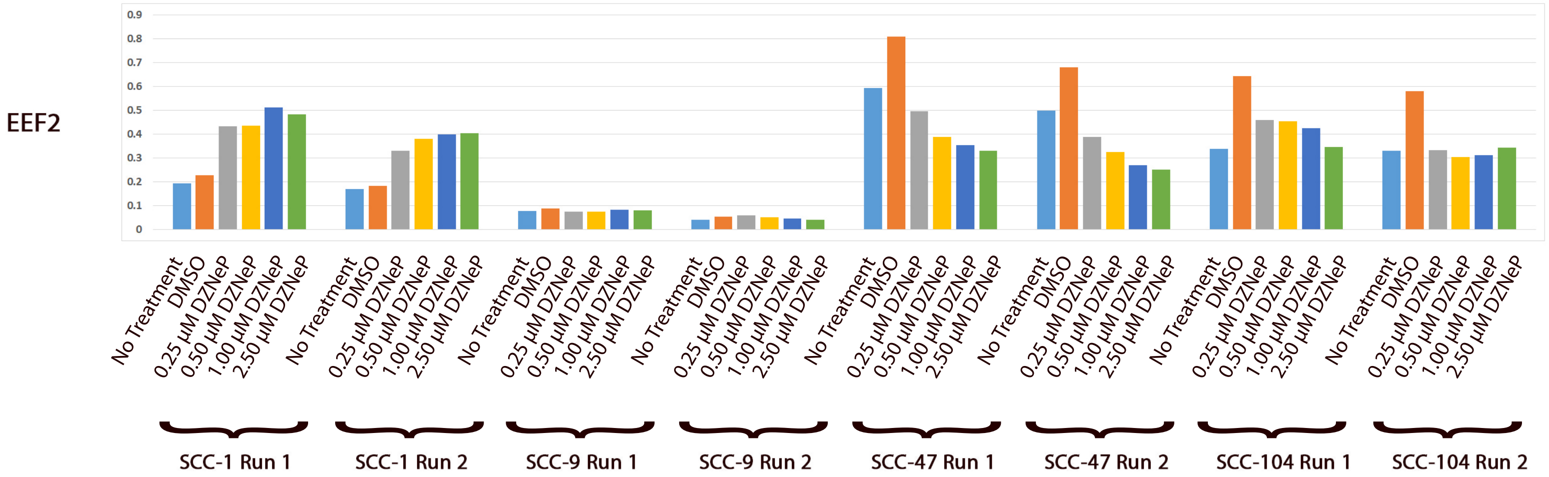

Supplemental Figure 10.
EGFR displays comparable trends in when both EEF2 and GAPDH are utilized as internal controls. Droplet digital PCR analysis of expressional ratios of EGFR utilizing either GAPDH (above) or EEF2 (below) following 7-day treatment with DZNeP. Scales vary according to individual expression results.
