# Supplementary figures and images for: Efficacy of EZH2 inhibitory drugs in human papillomavirus-positive and human papillomavirus-negative oropharyngeal squamous cell carcinomas
Source: Clin Epigenetics. 2017 Sep 6;9:95. doi: 10.1186/s13148-017-0390-y (PMC5586065; doi:10.1186/s13148-017-0390-y)

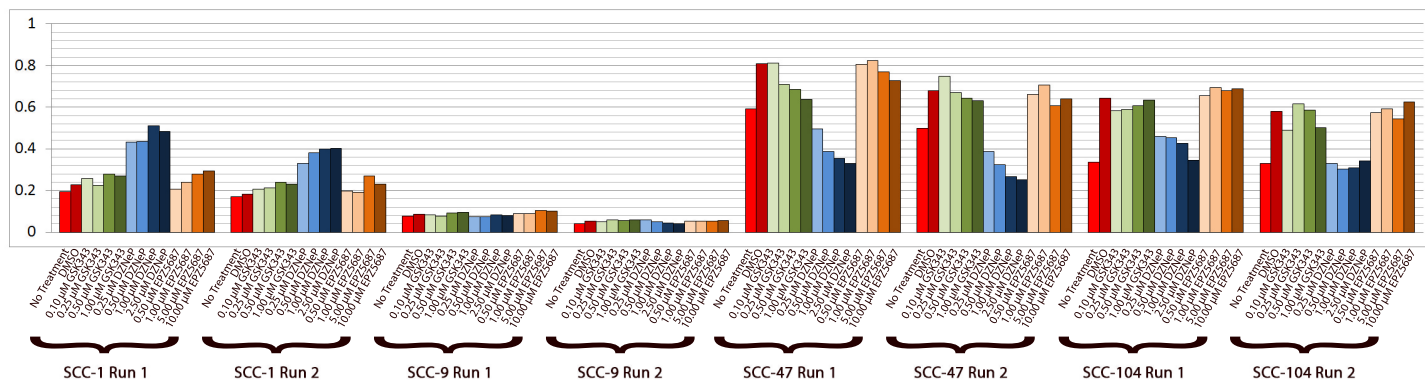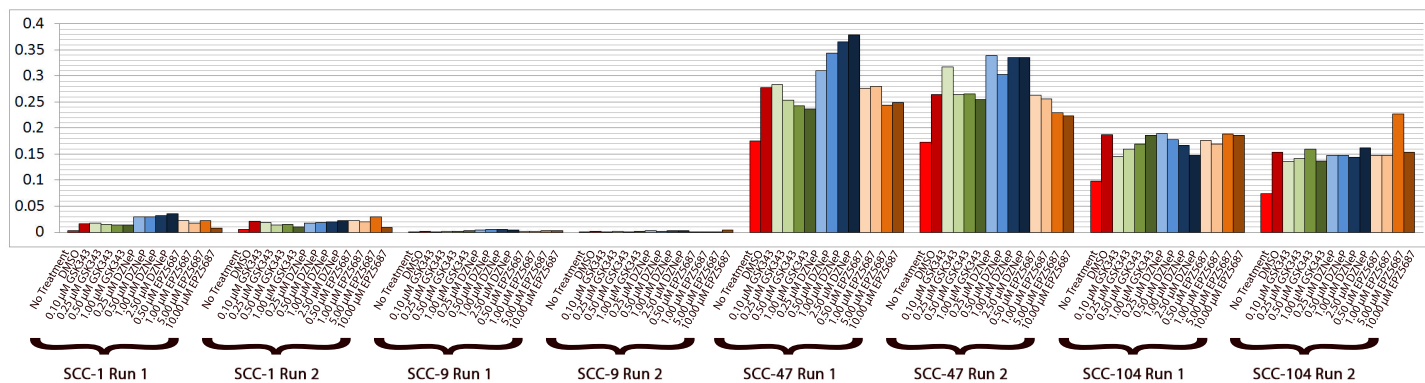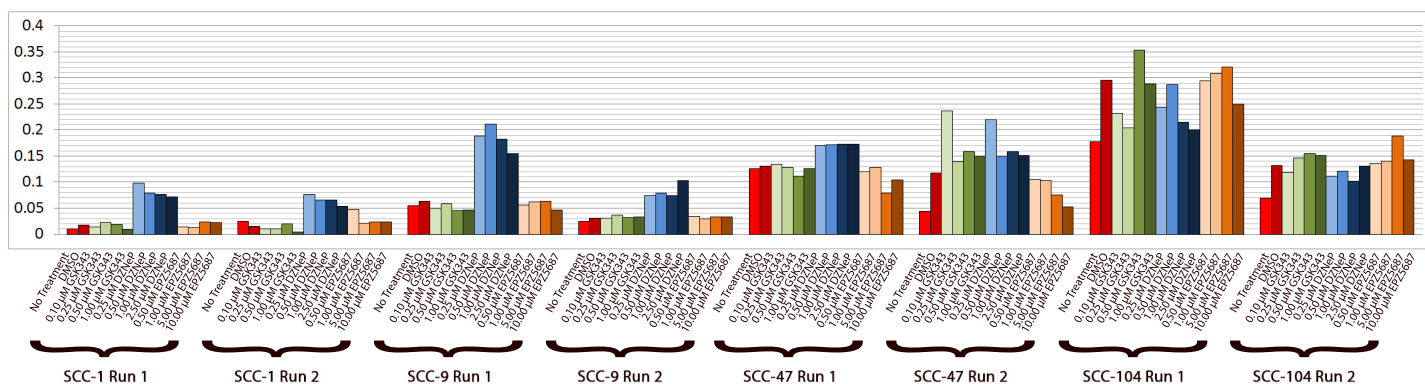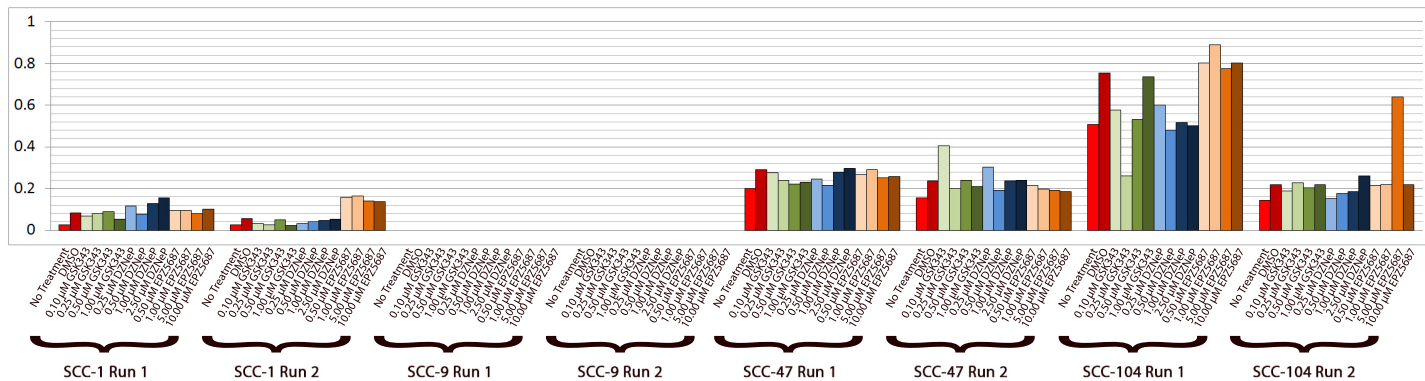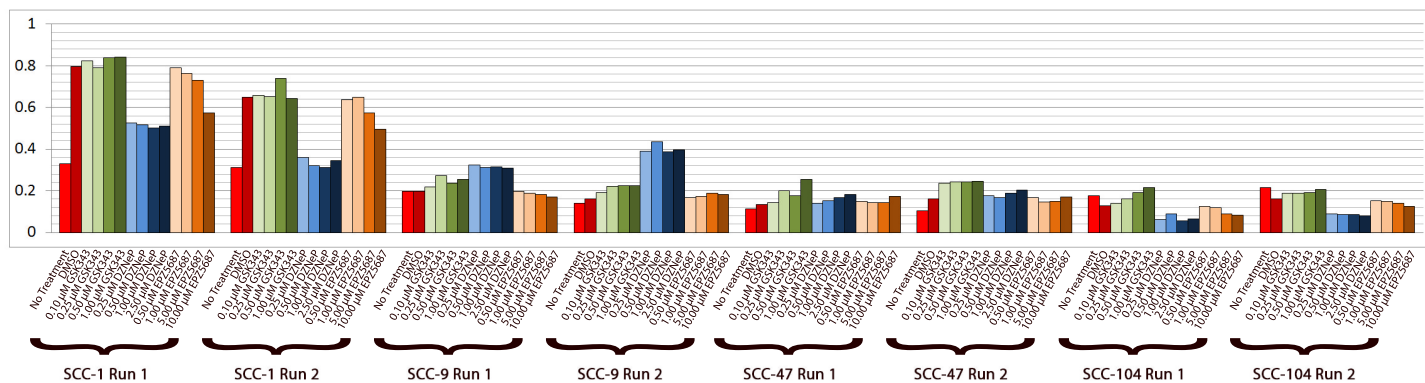

Supplement: Supplementary file 4 — Supplemental Figure 9. Treatment with inhibitors variably alters gene expression in all cell lines. (ZIP 148366 kb) [file 13148_2017_390_MOESM4_ESM.zip › Supp 9p1.pdf]
