# Supplementary material for: Efficacy of EZH2 inhibitory drugs in human papillomavirus-positive and human papillomavirus-negative oropharyngeal squamous cell carcinomas
Source: Clin Epigenetics. 2017 Sep 6;9:95. doi: 10.1186/s13148-017-0390-y (PMC5586065; doi:10.1186/s13148-017-0390-y)
Supplement: Supplementary file 5 — Supplemental Figures 1-4. Wound healing assay 0 hour to 72 hour timeline all time points. Wound made same day as treatment with inhibitor. (PDF 118729 kb) [file 13148_2017_390_MOESM5_ESM.pdf]

SCC-47

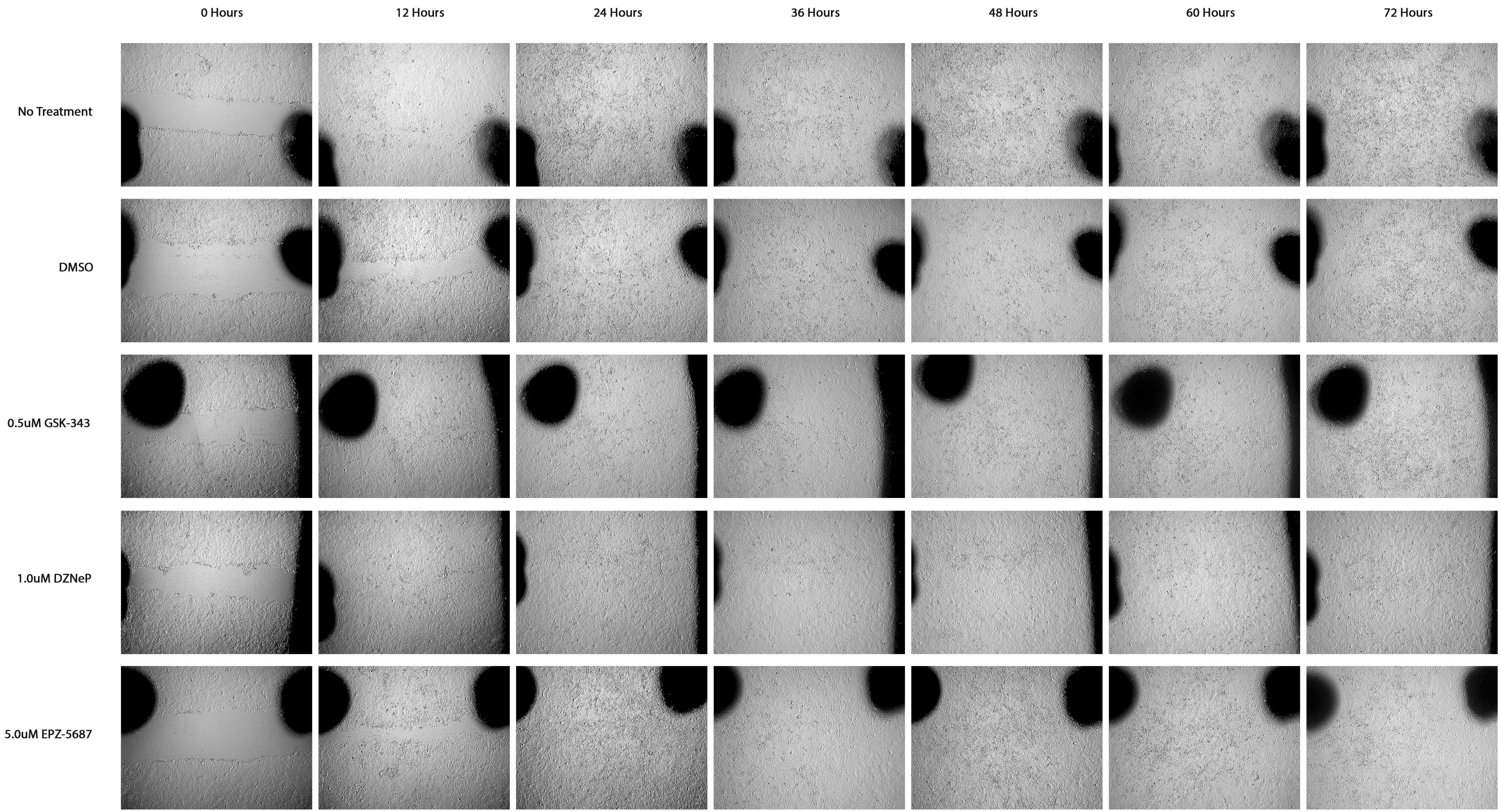

Supplemental Figure 1:
Wound healing assay SCC-47 0 hours to 72 hour timeline all time points. Wound made same day as treatment with inhibitor.

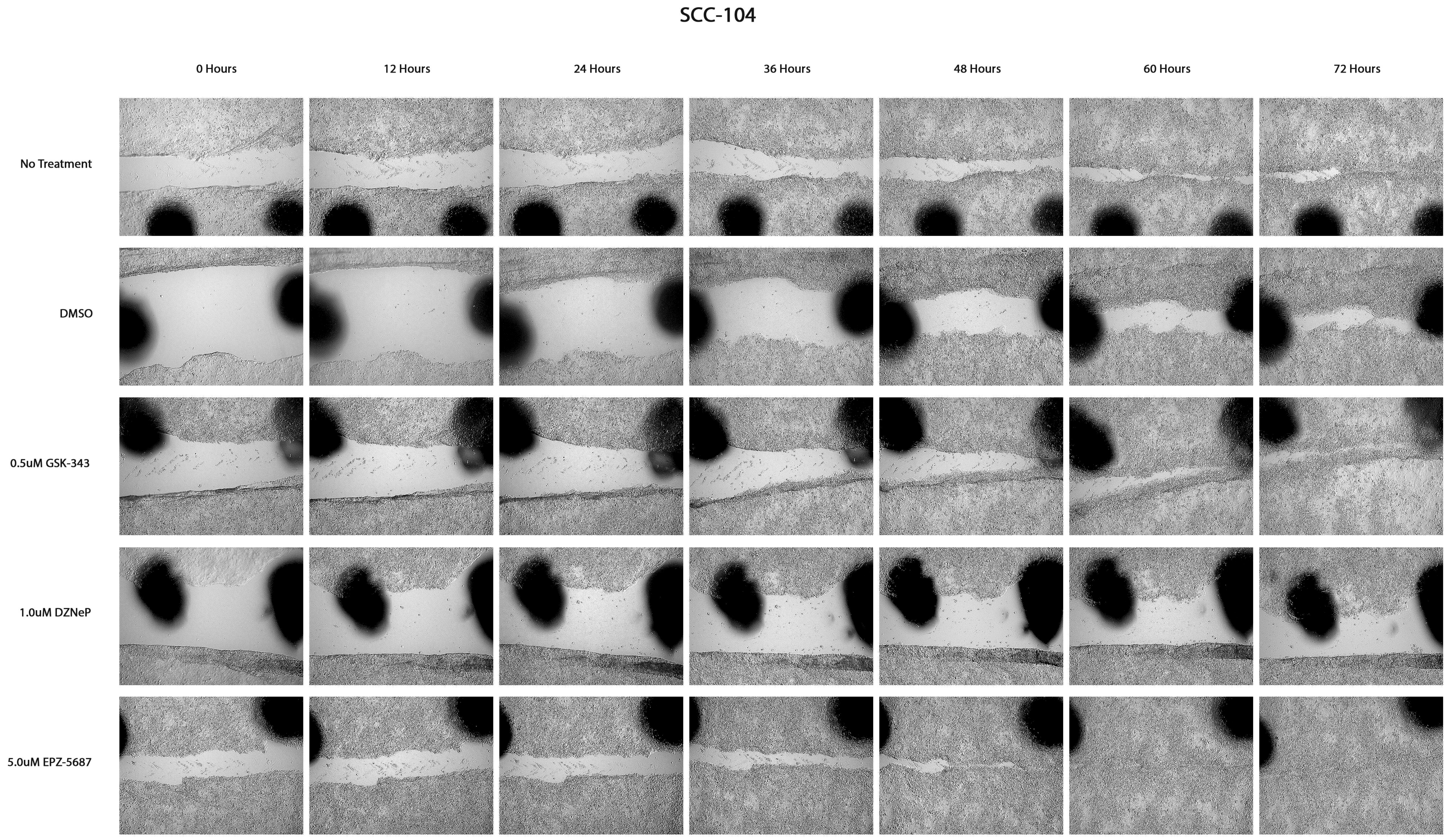

Supplemental Figure 2:
Wound healing assay SCC-104 0 hours to 72 hour timeline all time points. Wound made same day as treatment with inhibitor.

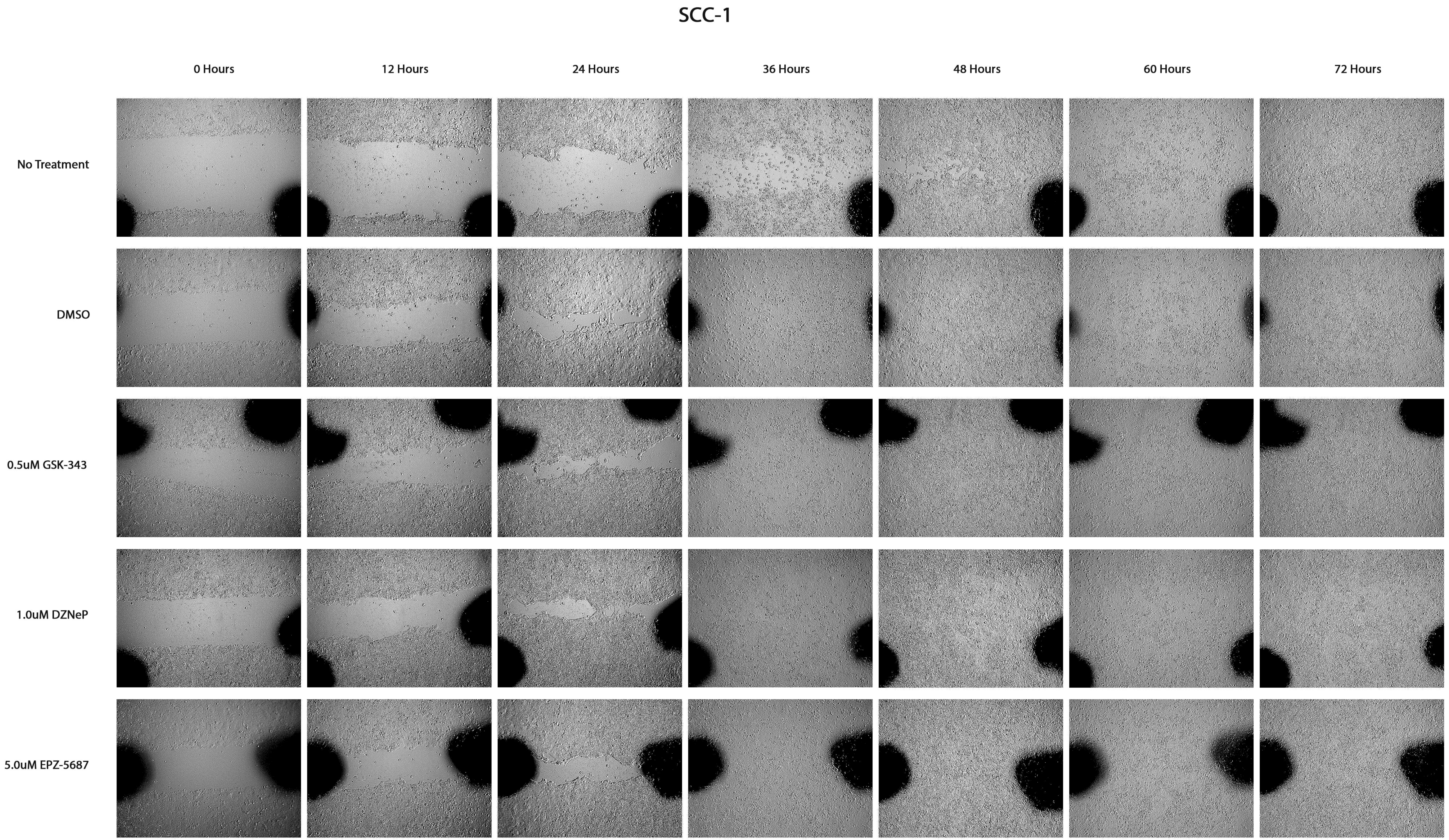

Supplemental Figure 3:
Wound healing assay SCC-1 0 hours to 72 hour timeline all time points. Wound made same day as treatment with inhibitor.

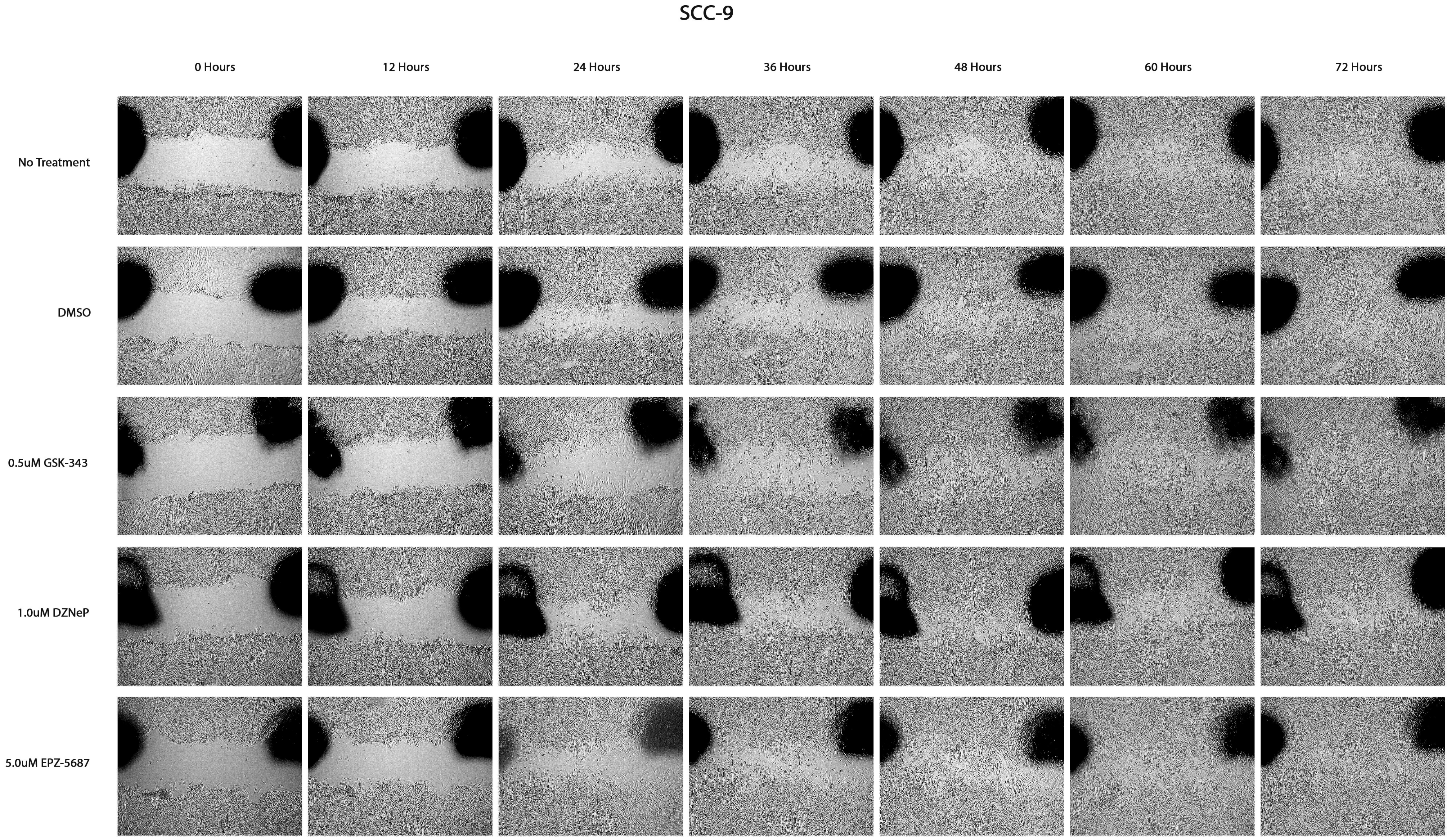

Supplemental Figure 4:
Wound healing assay SCC-9 0 hours to 72 hour timeline all time points. Wound made same day as treatment with inhibitor.
