# Supplementary material for: Efficacy of EZH2 inhibitory drugs in human papillomavirus-positive and human papillomavirus-negative oropharyngeal squamous cell carcinomas
Source: Clin Epigenetics. 2017 Sep 6;9:95. doi: 10.1186/s13148-017-0390-y (PMC5586065; doi:10.1186/s13148-017-0390-y)
Supplement: Supplementary file 6 — Supplemental Figures 5-8. Wound healing assay 0 hour to 72 hour timeline all time points. Wound made 5 days after treatment with inhibitor.(PDF 119715 kb) [file 13148_2017_390_MOESM6_ESM.pdf]

SCC-47

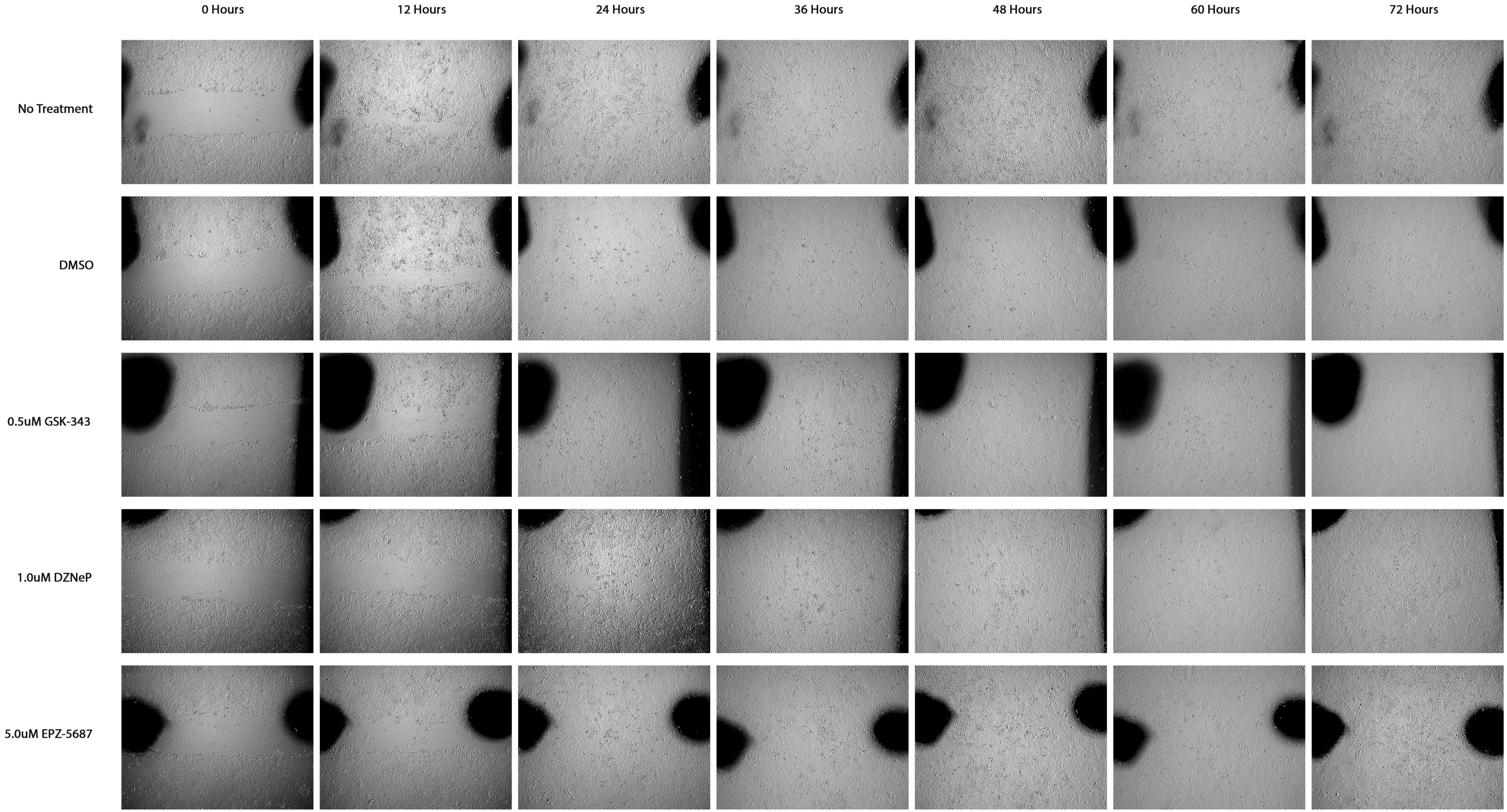

Supplemental Figure 5:
Wound healing assay SCC-47 0 hours to 72 hour timeline all time points. Wound made 5 days after treatment with inhibitor.

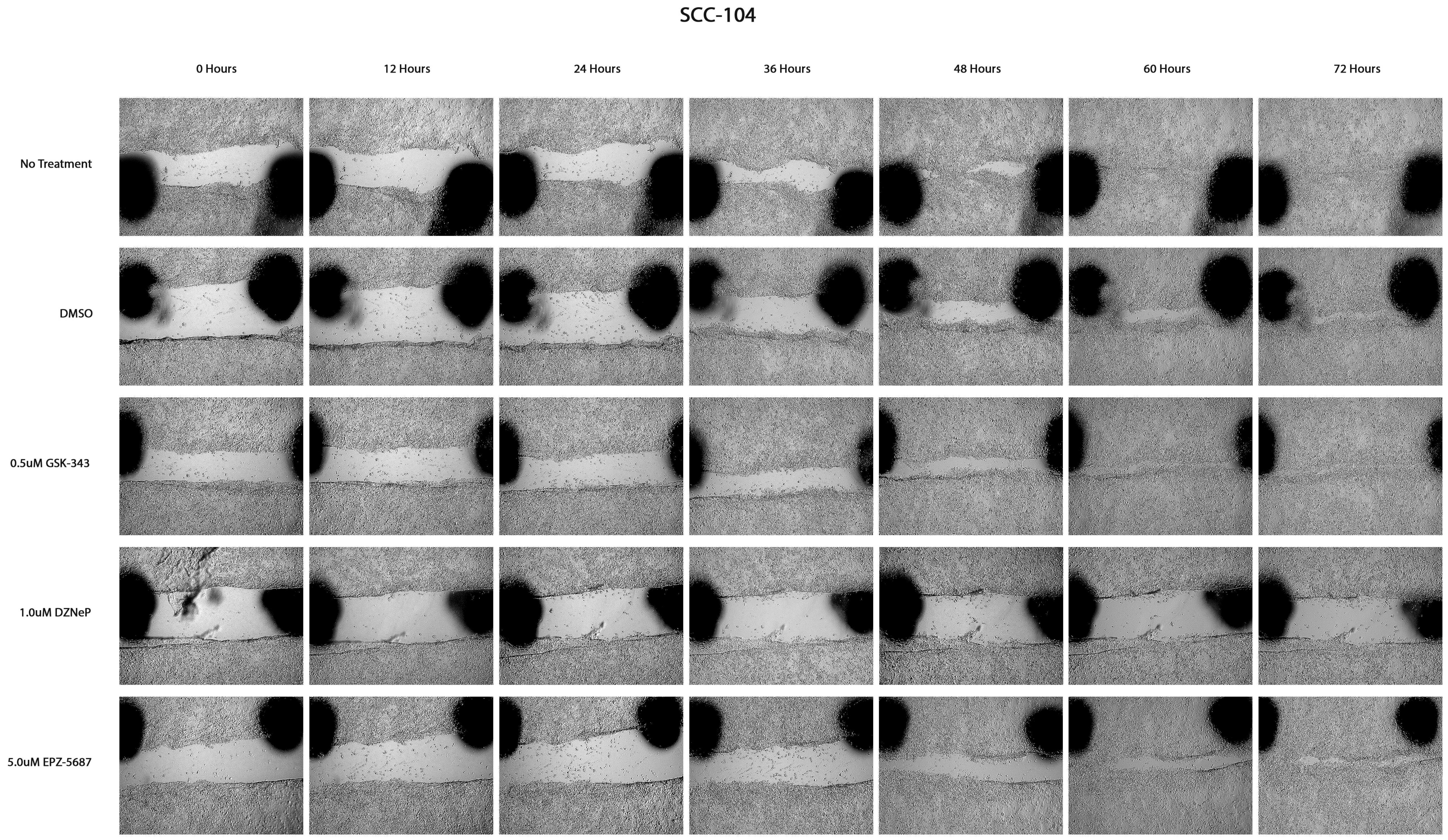

Supplemental Figure 6:
Wound healing assay SCC-104 0 hours to 72 hour timeline all time points. Wound made 5 days after treatment with inhibitor.

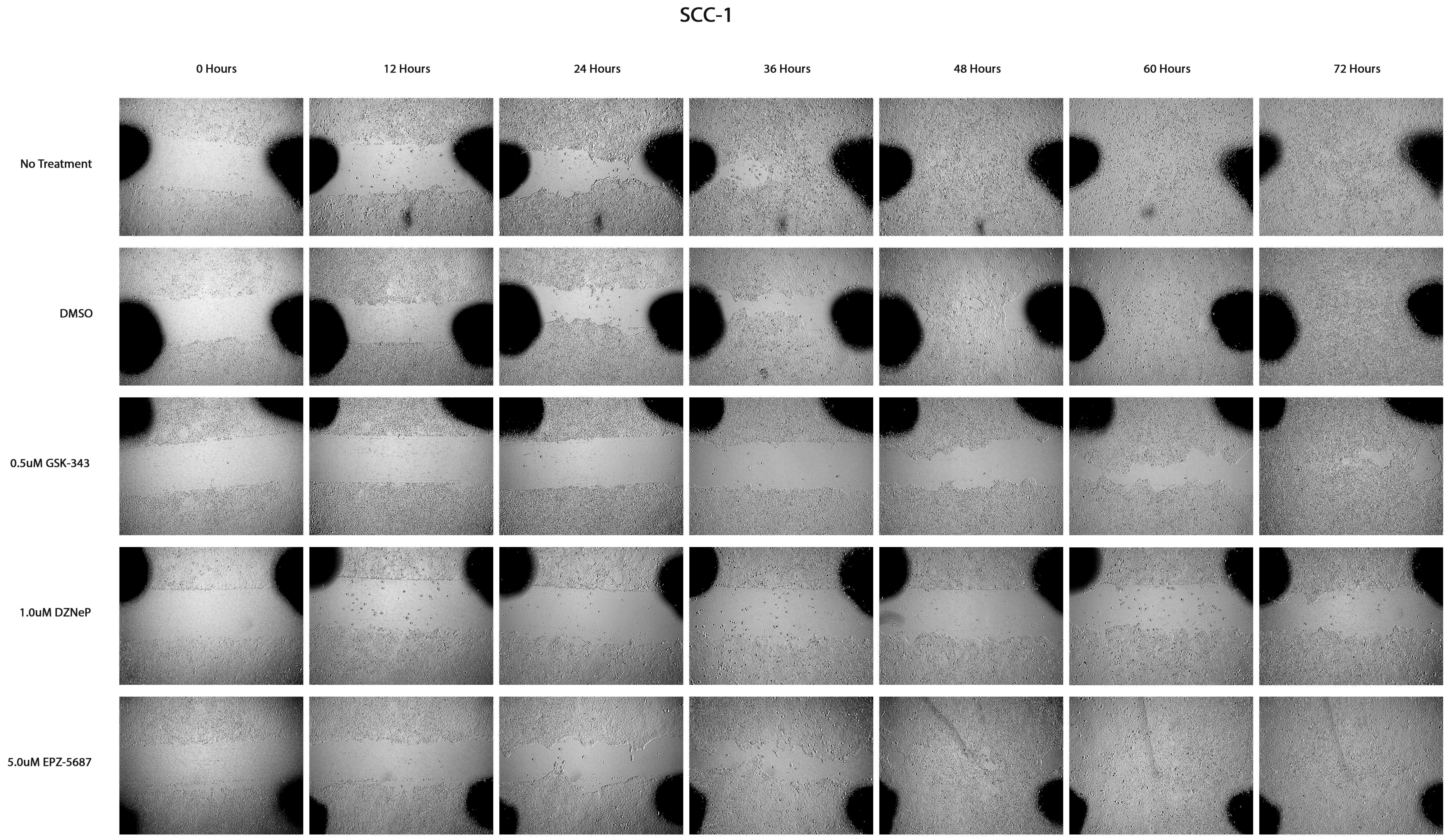

Supplemental Figure 7:
Wound healing assay SCC-1 0 hours to 72 hour timeline all time points. Wound made 5 days after treatment with inhibitor.

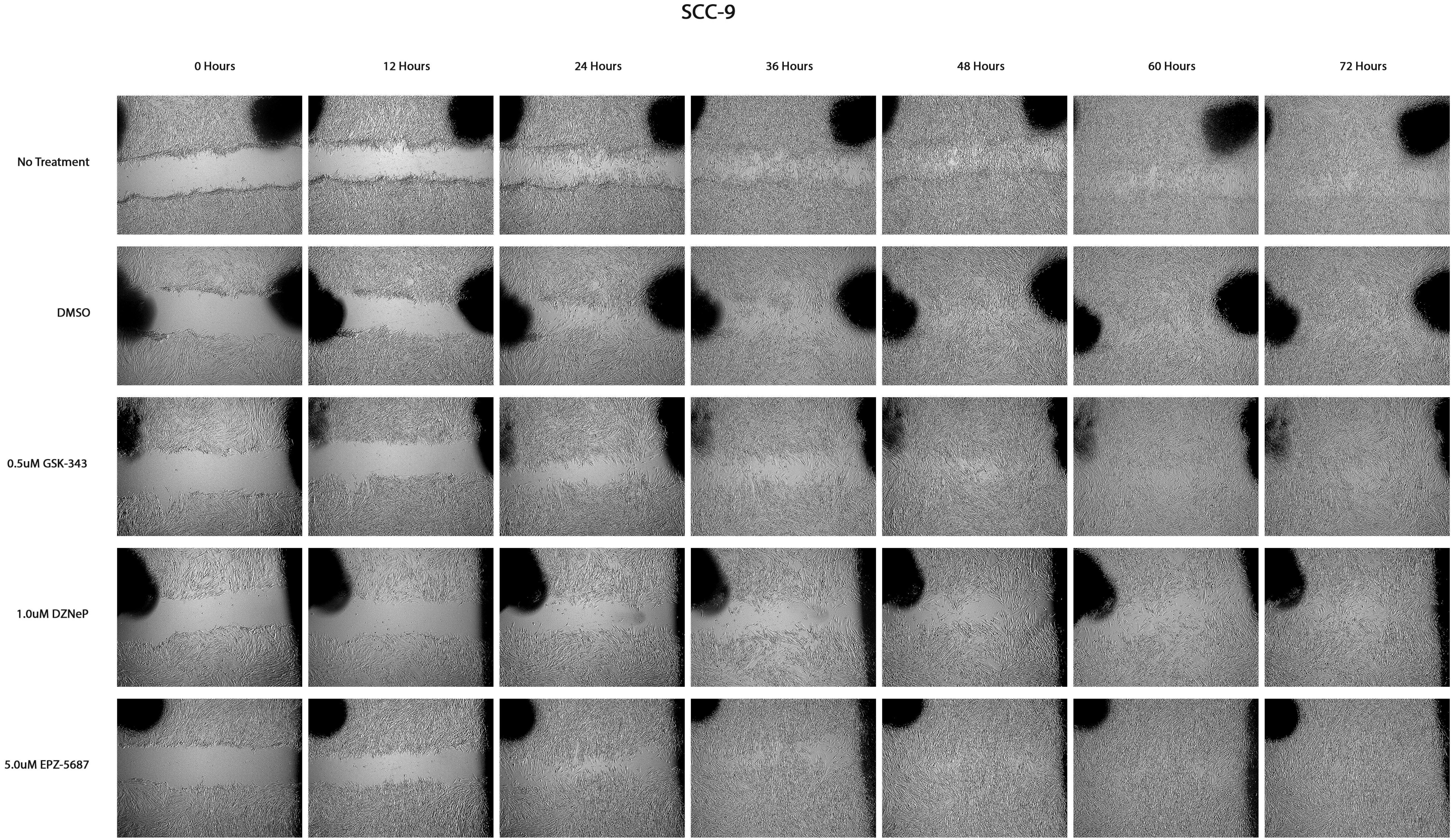

Supplemental Figure 8:
Wound healing assay SCC-9 0 hours to 72 hour timeline all time points. Wound made 5 days after treatment with inhibitor.
