# Supplementary material for: Efficacy of EZH2 inhibitory drugs in human papillomavirus-positive and human papillomavirus-negative oropharyngeal squamous cell carcinomas
Source: Clin Epigenetics. 2017 Sep 6;9:95. doi: 10.1186/s13148-017-0390-y (PMC5586065; doi:10.1186/s13148-017-0390-y)
Supplement: Supplementary file 2 — Supplemental Figure 12. Dimethylated H3K27 levels do not appear to change following treatment with inhibitors. (PDF 17232 kb) [file 13148_2017_390_MOESM2_ESM.pdf]

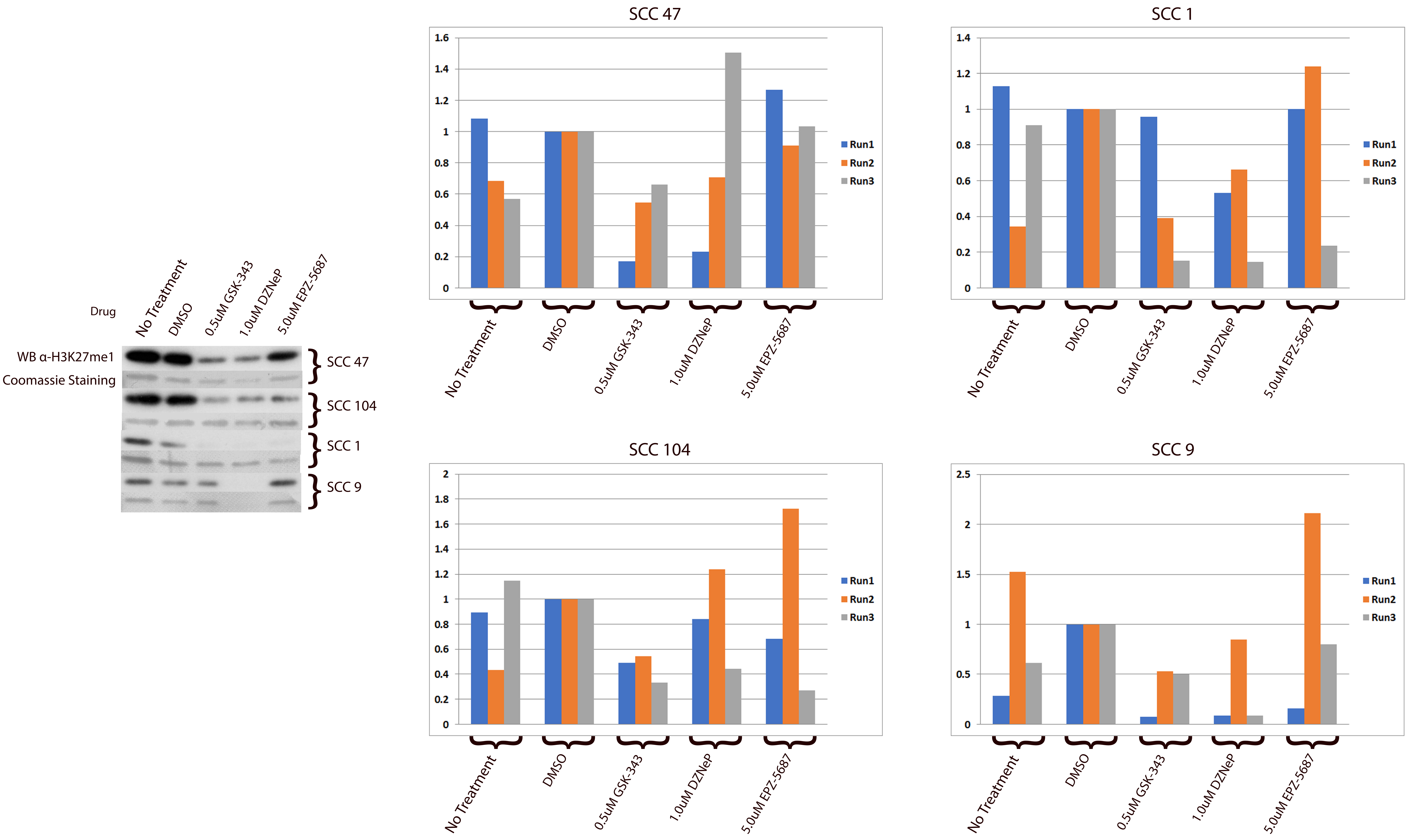

Supplemental Figure 12.
Dimethylated H3K27 levels do not appear to change following treatment with inhibitors.
Left: Western Blot analysis of H3K27me2 following 7-day treatments with GSK-343, DZNeP, or EPZ-5687. Run 3 pictured. Coomassie blue staining (shown below western blot) utilized as loading control. Right: Quantification of three runs of Western blots based on fold differences to DMSO-treated cells.
