# Supplementary material for: Efficacy of EZH2 inhibitory drugs in human papillomavirus-positive and human papillomavirus-negative oropharyngeal squamous cell carcinomas
Source: Clin Epigenetics. 2017 Sep 6;9:95. doi: 10.1186/s13148-017-0390-y (PMC5586065; doi:10.1186/s13148-017-0390-y)
Supplement: Supplementary file 4 — Supplemental Figure 9. Treatment with inhibitors variably alters gene expression in all cell lines. (ZIP 148366 kb) [file Supp 9p2.pdf › Supp 9p2.pdf]

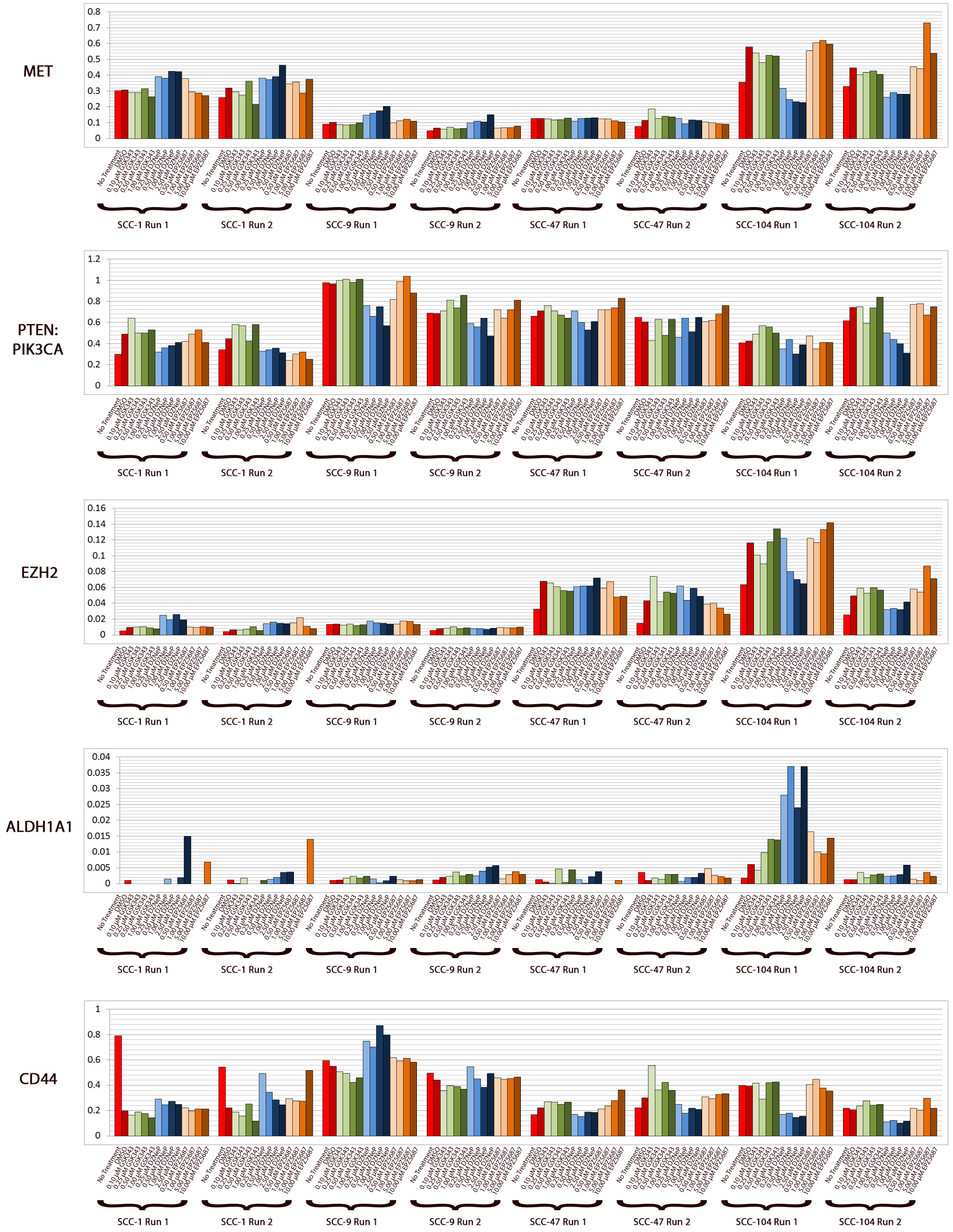

Supplemental Figure 9:
Treatment with inhibitors variably alters gene expression in all cell lines. Droplet digital PCR analysis of expressional ratios of 9 target genes (EGFR, TP53, MKI67, CDKN2A, CCND1, MET, PTEN, PIK3CA, EZH2, ALDH1A1, and CD44) following 7-day treatment with GSK-343, DZNeP, or EPZ-5687. EEF2 was utilized as an internal reference, with exception to PTEN:PIK3CA as their gene products are directly antagonistic to one another. Scales vary according to individual expression results. SCC-9 cell line does not express CDKN2A and is therefore not pictured.
